# Tunable x-ray free electron laser multi-pulses with nanosecond separation

**DOI:** 10.1038/s41598-022-06754-y

**Published:** 2022-02-28

**Authors:** Franz-Josef Decker, Karl L. Bane, William Colocho, Sasha Gilevich, Agostino Marinelli, John C. Sheppard, James L. Turner, Joshua J. Turner, Sharon L. Vetter, Aliaksei Halavanau, Claudio Pellegrini, Alberto A. Lutman

**Affiliations:** SLAC National Accelerator Laboratory, 2575 Sand Hill Road, Menlo Park, CA 94025 USA

**Keywords:** X-ray crystallography, Core processes, Lasers, LEDs and light sources, Optical techniques, Other photonics, Techniques and instrumentation

## Abstract

X-ray Free Electron Lasers provide femtosecond x-ray pulses with narrow bandwidth and unprecedented peak brightness. Special modes of operation have been developed to deliver double pulses for x-ray pump, x-ray probe experiments. However, the longest delay between the two pulses achieved with existing single bucket methods is less than 1 picosecond, thus preventing the exploration of longer time-scale dynamics. We present a novel two-bucket scheme covering delays from 350 picoseconds to hundreds of nanoseconds in discrete steps of 350 picoseconds. Performance for each pulse can be similar to the one in a single pulse operation. The method has been experimentally tested with the Linac Coherent Light Source (LCLS-I) and the copper linac with LCLS-II hard x-ray undulators.

## Introduction

With ten orders of magnitude brighter photon beams than third-generation light sources, the x-ray Free Electron Lasers (XFELs) are the brightest sources of x-rays for scientific applications^1–4^. The unique features of wavelength tunability, femtosecond pulse duration, and excellent transverse coherence are used in several fields of scientific investigation, including atomic, molecular, and optical physics, chemistry, biology, condensed matter physics, and matter in extreme conditions^5^. X-ray pulse customization has been a very active field of investigation, with the demonstration of novel ultra-short high power modes^6,7^, polarizaton control^8–10^ and two-color double pulses^11–18^. Double x-ray pulses were developed to perform x-ray pump/x-ray probe experiments, where ultra-fast physical and chemical dynamics, initiated by an x-ray pulse, can be explored by a second ultrashort x-ray probe pulse. Such pulses are routinely produced with a split undulator^11,16^, or with a twin-bunch technique^15^. The temporal separation between the pulses is limited to less than 125 fs with the Twin-bunch mode, and double pulses with a maximum delay of about 1 picosecond are routinely produced with the fresh-slice scheme^16^. However, there are experiments requiring longer temporal separation. As an example, the explosion of water droplets could be studied by triggering processes depending on pressure with a first x-ray pulse and then probing them with a second x-ray pulse nanoseconds later^19^. The filamentation effect in gas devices induced by x-rays can be probed with a second pulse with a delay of above 120 ns^20^. In the x-ray probe/x-ray probe class of experiments, neither pulse is meant to drive the sample to a different state, but the two x-ray pulses can be effectively compared after scattering and used to extract information at well-defined time separation. As an example, the equilibrium fluctuations of magnetic skyrmions were studied from the speckle patterns recorded as function of the time delay between two attenuated x-ray pulses in the nanosecond range^21–25^. Recently, with the advent of x-ray cavity based systems at LCLS, two- and many-pulse mode delivery became crucial^26,27^. The cavity-based XFEL (CBXFEL) project is currently relying on the 220 ns double pulse mode and the x-ray Laser Oscillator (XLO)^28^ will utilize trains of up to 8 pulses with 35 ns separation. A number of Matter under Extreme Conditions (MEC) experiments also require up to 8 x-ray pulses with $\le$ 1 ns separation which can now be delivered^29–31^.

In this paper, we provide complete description of a novel two-bucket scheme, operating at LCLS-I, and LCLS-II undulators with the copper linac^32–34^. We extended the x-ray pulse delay range far beyond 1 ps with two electron bunches accelerated in different Radio-Frequency (RF) buckets. With the existing S-band accelerating structures operating at a frequency of 2.856 GHz, the minimum temporal delay available is $\sim 350$ ps, corresponding to a single bucket separation. The delay can be controlled in integer bucket numbers, or in 350 ps steps up to hundreds of nanoseconds. Existing and planned high repetition rate FEL machines based on super-conducting accelerator technology will produce trains of photon bunches with a repetition rate of the order of a MHz, and therefore with a minimum distance between the XFEL pulses much longer compared to what is achievable with the presented scheme. A similar technique was demonstrated at FERMI to produce double electron bunches with a maximum separation of $\sim 2.5$ ns. However, the lasing process was limited to the extreme ultra-violet wavelengths. The experimental setup of the Two- and Many-bucket schemes, the physics involved, the performance achieved and future plans are discussed.

## Methods

### The machine layout

**Figure 1:**
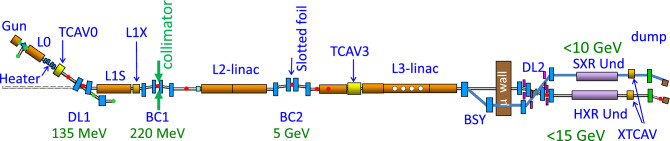
Schematic layout of the LCLS copper linac configuration. Electron bunches are extracted at a normal conducting gun and subsequently accelerated in Linac sections and compressed in the first (BC1) and second (BC2) bunch compressors. A laser heater increases the electrons energy spread to suppress a microbunching instability. X-band linearizer L1X provides longitudinal time-energy electron bunch control for improved bunch compression and reduces phase space nonlinearities. Transverse deflecting cavities (XTCAV) are mainly used for diagnostic purposes^35^. In the beam switchyard (BSY), the electron bunches are directed to either SXR or HXR undulator lines. In the undulator lines, the electron bunches produce x-ray Free-electron laser pulses. Downstream from the undulator, the electrons are discarded after a vertical bend in the beam dumps, while the x-rays are delivered to the experimental stations.

The copper LCLS XFEL is schematically represented in Fig. 1. It is able to deliver x-rays in the energy range between 280 eV and 20 keV. An electron bunch with a typical charge of 250 pC is extracted from a copper photocathode by a UV driving laser. There are currently two independent UV driving lasers in the LCLS photoinjector. The electrons are accelerated in a short linac section to 135 MeV and then travel through the laser heater section. The laser heater increases the electron bunch’s uncorrelated energy spread to prevent a microbunching instability from forming in the magnetic bunch compressors. The heater is composed of a four dipole chicane, a permanent magnet undulator, and an infrared (IR) laser. An energy modulation is impressed on the electrons co-propagating with the IR laser in the undulator. The last two dipoles of the chicane provide temporal smearing of the modulation, leaving increased energy spread within the bunch. After the first dogleg (DL1), the electrons are further accelerated to 239 MeV. The linac phase is adjusted to induce an energy chirp on the electron bunch suitable for bunch compression. Before entering the first bunch compressor (BC1), an X-band linearizer (L1X) is used to remove the second order energy chirp induced in the accelerating section, add more linear chirp, and decelerate the beam to 220 MeV. BC1 compresses the bunch to a current of 100 - 220 A in a typical operation. A collimation system is used to tailor the longitudinal current profile at the first bunch compressor^36^, typically reducing the charge to 180 pC. A 320 m long section of Linac (L2) increases the electron bunch energy to either 3 or 5 GeV. The second bunch compressor (BC2) compresses the electron bunch to its final duration, ranging from 30 to a few hundred femtoseconds at nominal charge operation. The last 540 m long linac section (L3) accelerates (or decelerates) the electron bunch to the final energy required for operation, from 2.5 to 17 GeV.

The electron bunch reaches the undulator hall entrance after a second dogleg (DL2) and a transport line. The high current, high brightness electron bunch lases in either the new 140-m long HXR undulator or in the new 78 m long SXR undulator^37^. We refer the reader to Ref.^38^ for the old LCLS-I fixed gap undulator line details. Downstream of the undulators, the electron bunch is directed into the beam dump, while the x-rays are sent to the experimental stations. X-ray diagnostics that are relevant for this work are gas energy monitors, which measures the pulse energy from the fluorescence yield of x-rays traveling in a gas cell, a YAG screen used to destructively measure the x-ray’s transverse pulse profile at LCLS-I, and a Micro-Channel Plate (MCP) detector used to measure the pulse energy after a monochromator at longer wavelengths at LCLS-I, and a fast diode was/will be used for intensity measurements using harder x-rays at LCLS-I/LCLS-II.

The two-bucket mode with nanosecond separation was conceived and demonstrated at the LCLS-I^1^. As part of the LCLS-II upgrade, two new undulator beamlines have been installed while maintaining the operation with the existing copper linac. To enable operation in two-bucket mode, two independent UV drive lasers extract two separate electron bunches, with a time delay of multiples of 350 ps, suitable for the acceleration in two different RF buckets. The 350 ps is fixed by the S-band linac accelerating structure frequency of 2.856 GHz. The IR lasers in the laser heater section were also duplicated to ensure that the microbunching instability is mitigated for both electron bunches at the second bunch compressor. At BC1, the collimation of head and tail current spikes can be used only as long as both electron bunches travel in the bunch compressor at the same energy. Experimental setups requiring double pulses with an energy separation and short time delays need to be accelerated on different RF accelerating phases in the linear accelerator, thus preventing the use of the bunch collimation. For short delay separations ($\sim < 30$ ns), the RF pulses in the linear accelerator are sufficiently equal in phase and amplitude to operate with both bunches. However, for long delay separations ($\sim > 100$ ns), a modification of the RF station input waveforms is required.

### Two-bucket setup

**Figure 2:**
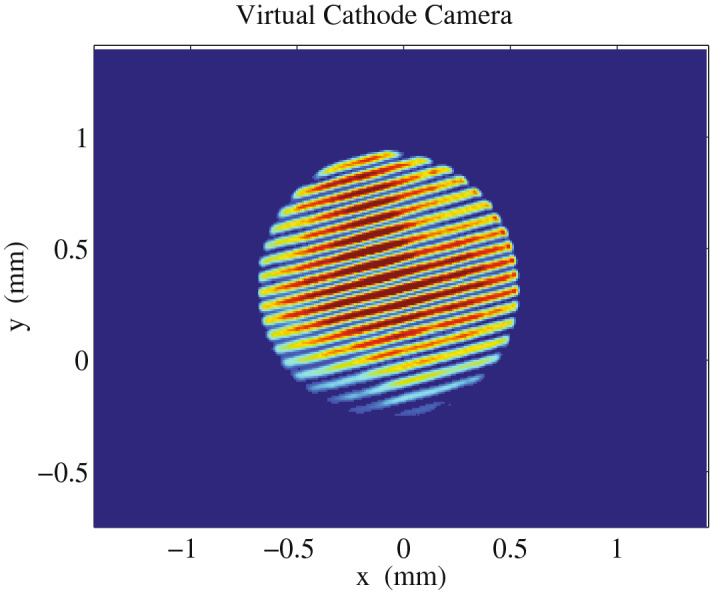
Interference fringes at the Virtual Cathode Camera when the two UV drive lasers are overlapped in time.

In a two-bucket FEL, both electron bunches lase in the same undulator line, using the same undulator segments. Therefore, particular care is required that the two electron bunches are as identical as possible at the undulator entrance in terms of the current profile, transverse matching and transverse orbit. Initially, the regular Self-Amplified Spontaneous Emission (SASE) beam is set up using one of the UV drive lasers to extract a single electron bunch at the photocathode. The longitudinal electron bunch phase space, the transverse matching and orbit are manipulated to optimize the lasing process. In such conditions, the electron bunch travels on the optimal orbit in the machine. The orbit of the first bunch can be optimized with the standard procedure. The second bunch is set to operate at the same extraction phase of the first. To this aim, the second drive laser timing is set to the same RF bucket and its temporal delay is scanned to maximize an interference pattern measured at the virtual cathode camera, obtaining a pattern like the one shown in Fig. 2. The amount of charge extracted can be controlled by rotating a waveplate and the charge balance can be verified by alternatively shuttering the drive lasers.

**Figure 3:**
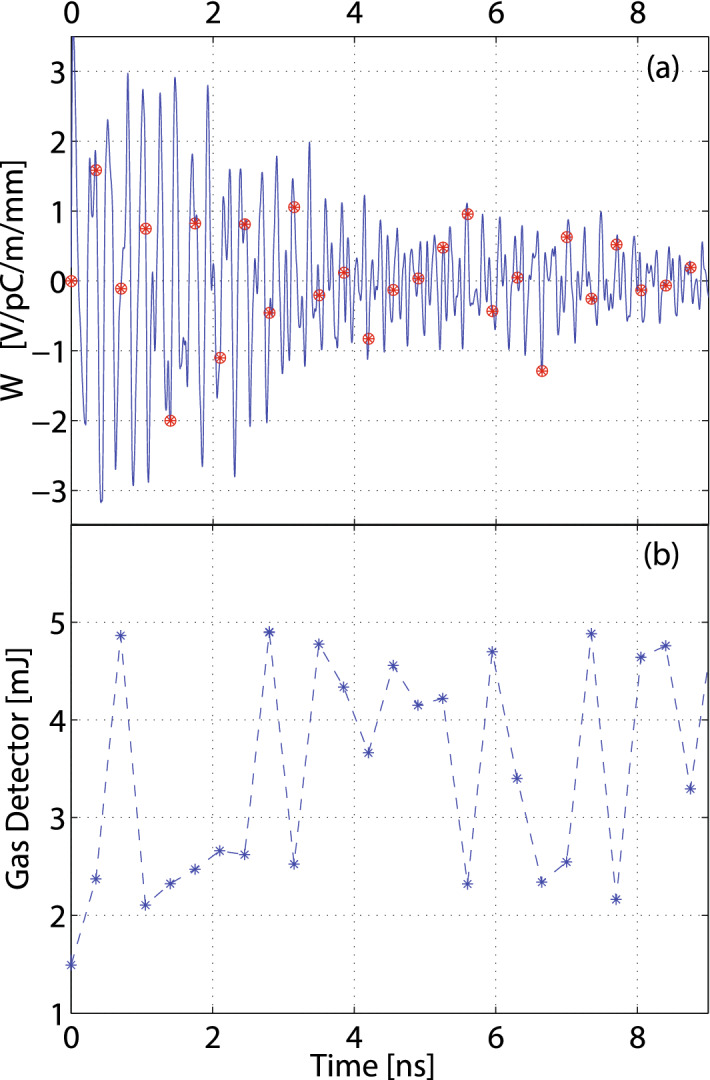
Transverse wakefields and their impact on the lasing process for short delays: (**a**) long-range transverse wakefield for the S-band accelerating structure. Red dots represent 350 ps sampling, (**b**) typical LCLS-I lasing energy performance as a function of the two-bucket delay (with no active trajectory correction).

The most significant challenges for short time delays of less than $\sim 25$ ns are the interaction between the bunches through wakefields and the interpretation of the transverse Beam Position Monitor (BPM) diagnostics. The long-range wakefields are relevant for the two-bucket scheme, because the transverse wakefield kick imparted by the leading bunch onto the trailing bunch can prevent it from lasing in the undulator line. The lasing suppression by orbit is routinely used in Fresh-slice operation^16^, or to collect baseline images for the x-ray temporal reconstruction based on transverse deflecting cavities^39^, but it is an unwanted effect in two-bucket operation. The long-range single- and multi-bunch transverse wakefields have been studied theoretically for the S-band accelerating structures and for the X-band linearizer structure^40,41^. The transverse wakefields of the linearizer are $\sim 20$ times stronger per unit of length compared to those of the accelerating structures up to distances corresponding to one and two bucket separation. However, since the accelerating structures are much longer, the main contribution to the transverse wakefields is due to the S-band accelerating structures. In Fig. 3a, the blue curve represents the calculated transverse, long-range wakefield for the copper S-band accelerating structures, while the red dots are the discrete 350 ps sampling points. Figure 3b shows the measured total pulse energy produced in the two-bucket mode as a function of the delay at an energy of 8.8 keV. The bucket delay was scanned with the transverse orbit feedback turned off, therefore the first bunch was lasing by travelling on the design orbit, while the trailing bunch was subject to different transverse wakefields as a function of the bunch delay. The first point corresponds to both drive lasers in the same bucket. For delays with a strong wakefield interaction the pulse energy is down to $\sim 2.5$ mJ produced exclusively by the first bunch, with a maximum total of $\sim 5$ mJ in case of two bunches lasing equally. To cancel the transverse wakefield adverse effect at a specific RF bucket separation, electron bunch orbit bumps or oscillating orbits can be introduced at particular locations in the linac to recover the full performance.

Another obstacle to overcome in the two-bucket mode is measuring the electron bunch orbit precisely. Several types of BPMs are used to record the electron bunch orbit in the LCLS and were designed to operate with a single electron bunch. In two-bucket mode, the measurement of the transverse position fails for particular bunch delays, depending on the BPM type. For example, the LCLS-I stripline BPMs in the Linac fail with a periodicity of $\sim 7$ ns because of a 140 MHz narrow bandpass filtering. When an operation requires two-bucket pulses with such delays, the feedbacks requiring problematic BPMs must be turned off. Therefore the machine has to be finely re-tuned, by switching back to single bunch mode, every few minutes of operation to avoid performance degradation. Stable performance is instead ensured by the feedbacks for delays where the BPMs work properly.

For long delays (roughly above 100 ns), a special setup is required for each RF station, otherwise the amplitude and phase are not sufficiently equal for both pulses. The standing wave RF gun increases its field exponentially for a flat input pulse. Therefore after an initial rise time, the input pulse needs to be lowered to keep the field in the gun cavity constant. However, due to the presence of a reflected RF pulse travelling back to the gun, the input RF pulse must be also tailored with a counteracting input pulse with a specific amplitude and phase offset. With this expedient, a flat pulse of $\sim 500$ ns can be achieved. Unfortunately, the reflected part drifts over half-day long time-scales by about $2\%$, most likely due to temperature changes. The accelerating structures upstream of BC1 require a lengthening of the input pulse. The linearizer X-band cavity L1X input pulse cannot be simply lengthened to cover both pulses because it would lead to excessive power dissipation. Instead, the input pulse has been doubled, with the second one delayed to cover the second pulse. The phase of the second pulse is then finely tuned to achieve the same compression for both bunches in BC1. The Linac sections downstream of BC1 routinely run in SLAC Linac Energy Doubler (SLED) mode^42–44^. In SLED mode, an initial $4\mu$s RF pulse is compressed to a length of 825 ns FWHM corresponding to the accelerator fill-time, increasing the operating LCLS range from $\sim 6$ keV to 13 keV. Producing two-bucket pulses at harder x-rays and long delay times requires the use of the SLED mode. The electron bunches need to be carefully placed, one on the rising side and one on the falling side of the RF pulse, to achieve the same final energy. However, running off-crest decreases the maximum energy gain achievable in the Linac. For the L3 section, running at a maximum of 825 ns separation decreases the energy gain from 10 to 7 GeV, thus limiting the operation at shorter wavelengths. Electron bunches with a temporal separation of up to 700 ns have been produced by running without the SLED mode, and lasing capabilities have still to be tested for such long delays.

### Longitudinal phase-space tuning

**Figure 4:**
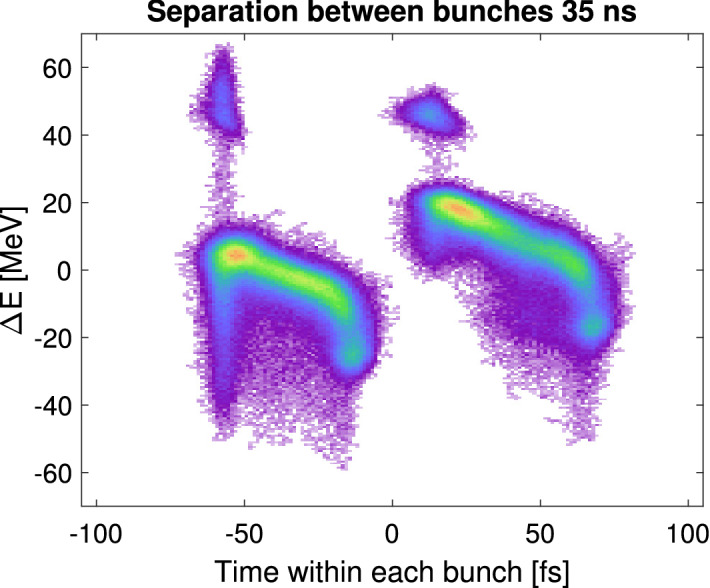
XTCAV image of the two-bunch beam with 35 ns separation (overlapped on the same fs scale). Leading (right) bunch produced about 0.5 mJ and the trailing (left) bunch lased at about 2 mJ at 9 keV photon energy.

Final longitudinal phase space adjustments are made using the transverse deflecting cavity (XTCAV) to observe the bunch differences. In order to minimize the chirp difference between the two bunches, L1X and L2 phases are fine-tuned. Best performance in two-bunch mode is achieved when the two bunches are laser heated. Fig. 4 shows an example XTCAV image of two laser heated electron bunches spaced by 35 ns.

### Two-bucket jitter

An important experimental parameter is temporal and energy jitter in the two-bucket mode. Measurements of the relative two bunch time jitter were done for different separations. For 50 ns bunch separation we found the timing jitter to be about 6 fs RMS and the energy jitter to be 2 MeV RMS; for 220 ns separation the jitter was 6.5 fs RMS and the energy jitter 5.5 MeV RMS. Currently, efforts are underway to minimize the energy jitter in the two bucket mode. For short inter-bunch spacings the temporal and energy jitter values are similar to the single bunch mode.

### Many-bucket setup

In order to produce more than 2 x-ray pulses, we implemented a UV pulse stacker^34^, which is shared with the two UV drive lasers. Current system setup allows for up to 8, evenly-spaced buckets, with short (about 2 ns) inter-bunch delays and moderate XFEL performance, or two groups of up to 4 pulses spaced with an arbitrary delay; see^34,45,46^. Achieving more than 2 ns between the pulses is limited due to real-estate constraints of the pulse stacker. In the multi-bucket configuration, cascade wakefield effects may become important and prevent the bunch train from lasing^41^. Another complication is the measurement and control of the multi-bunch trajectory. We summarize the results of LCLS-I and LCLS-II experiments in the sections below. In both cases, two- and multi-bucket lasing suffered from e-beam bunch-by-bunch trajectory differences. These differences are exacerbated for bunch separations of >30 ns. An effort is currently underway to implement an ultra-fast stripline kicker system in the copper linac to perform bunch-by-bunch correction^47,48^. We also point out that currently, electron bunches can be laser-heated only in single and two-bucket modes. An upgrade to the laser infrastructure is being considered to allow for multi-bunch laser-heating.

## Results

### LCLS-I results

**Figure 5:**
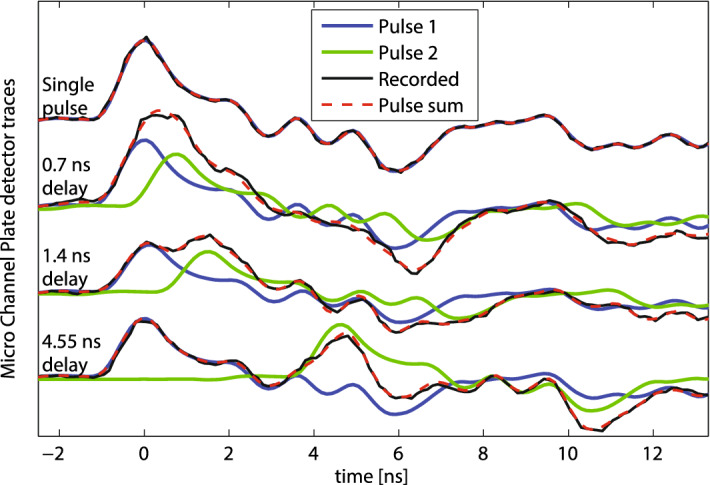
Micro-Channel Plate traces for different two-bucket delay configurations in the short delay regime. The recorded signal (black) is decomposed in the sum (dashed red) of two contributions, the leading pulse (blue) and the trailing pulse (green). The recorded signal has been filtered in the frequency domain removing components corresponding to 1, 2 and 3 samples separation.

The two-bucket scheme was first demonstrated in the Soft x-rays at 1195 eV, operating with a total charge after collimation of 410 pC, and a bunch current of 1.5 kA, with delays of up to tens of nanoseconds^21,49,50^. For such delays the gas energy detector measures the total pulse energy but cannot discriminate between the two pulses. The analysis of the MCP detector signal, located downstream of the Soft x-ray monochromator^51,52^, can recover the pulse intensity for each of the monochromatized beams. Figure 5 shows MCP traces for a single pulse and a double pulse operation with delays up to 4.5 ns separation. The linearity of the MCP signal and the well-defined delay between the pulses, allow for the use of a simple non-iterative algorithm to recover the contribution of each pulse starting from the measured single pulse response function.

**Figure 6:**
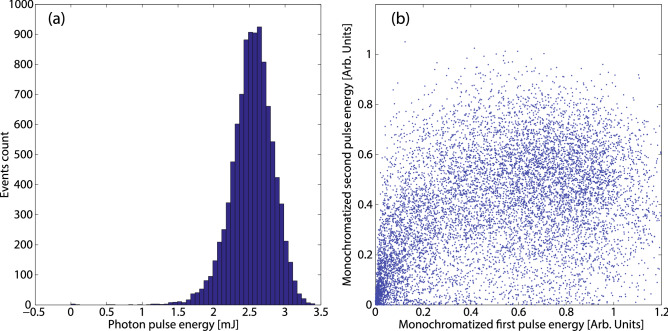
(**a**) Pulse energy histogram for two-bucket double pulses before monochromator. (**b**) Intensity of the first pulse vs intensity of the second pulse on 10000 consecutive shots after the monochromator.

For the 4.55 ns delay case 10000 consecutive shots were analyzed. Before monochromatization, the pulse energy was measured by the gas detectors to be $2.54 \pm 0.29$ mJ. The statistical distribution of the recorded energies is represented by the histogram in Fig. 6a. The intensity of the first and second pulses after the monochromator are shown in Fig. 6b. The fluctuations of each pulse increase after the narrow bandwidth monochromator as expected^53^, and the intensities of the pulses are randomly scattered. Experiments requiring pulses with controlled intensities after the monochromator require a reliable intensity measurement at the MCP while sorting the pulses on a shot to shot basis. For the considered set, 896 shots had intensities within 10% difference, and 1791 shots had intensities within 20%. The ability of measuring the relative pulse intensity after a monochromator is a relevant diagnostic to balance the double pulses enabling the optimization of several machine parameters. The performance achieved was similar for the other reported delays. The trailing x-ray pulse can be easily turned off by shuttering the driving laser, thus removing the second electron bunch. The first pulse can be selectively suppressed by increasing the IR laser heater power, so that the large energy spread prevents lasing in the undulator. The principle is the same as is used in the heater optical shaping^54^.

**Figure 7:**
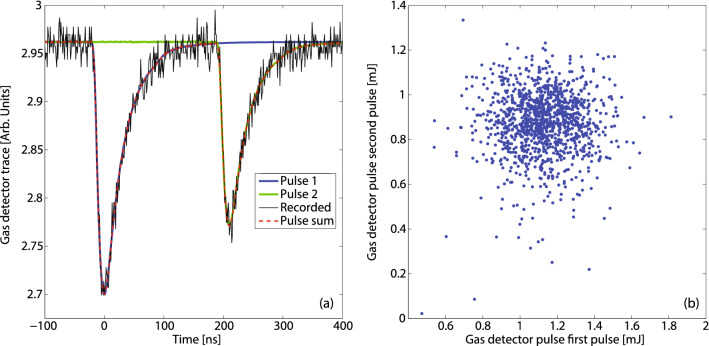
(**a**) Single-shot gas monitor detector traces for a two-bucket configuration with 210 ns delay (CBXFEL-like case). The recorded signal (black) is decomposed in the sum (dashed red) of two contributions, the first pulse (blue) and the trailing pulse (green). The first pulse energy was measured to be 1.25 mJ, and the second pulse energy was measured to be 0.91 mJ. (**b**) Intensity of the first pulse versus intensity of the second pulse on 1200 consecutive shots.

Figure 7a shows the raw gas detector signals for a two-bucket mode operating at an electron energy of 13.45 GeV, at a photon energy of 8.192 keV. Each electron bunch had a charge of approximately 180 pC after collimation, and a current of 3.2 kA. The electron bunch time separation was set to 600 RF buckets corresponding to 210 ns. The contribution of each pulse can be calculated by the time-resolved fluorescence yield for delays larger than a few tens of nanoseconds. In the figure, the full raw signal (black) is decomposed in the blue signal for the front pulse, with energy of 1.25 mJ and the green signal from the trailing pulse with an energy of 0.91 mJ. Figure 7b shows the intensity of the first pulse vs the intensity of the second pulse on 1200 consecutive shots measured by the gas detector. The first pulse had average energy of 1.12 mJ with fluctuations of 15%. The second pulse had average energy of 874 $\mu$J with fluctuations of 16%. The intensity of the two pulses was uncorrelated.

### New LCLS-II undulator results

**Figure 8:**
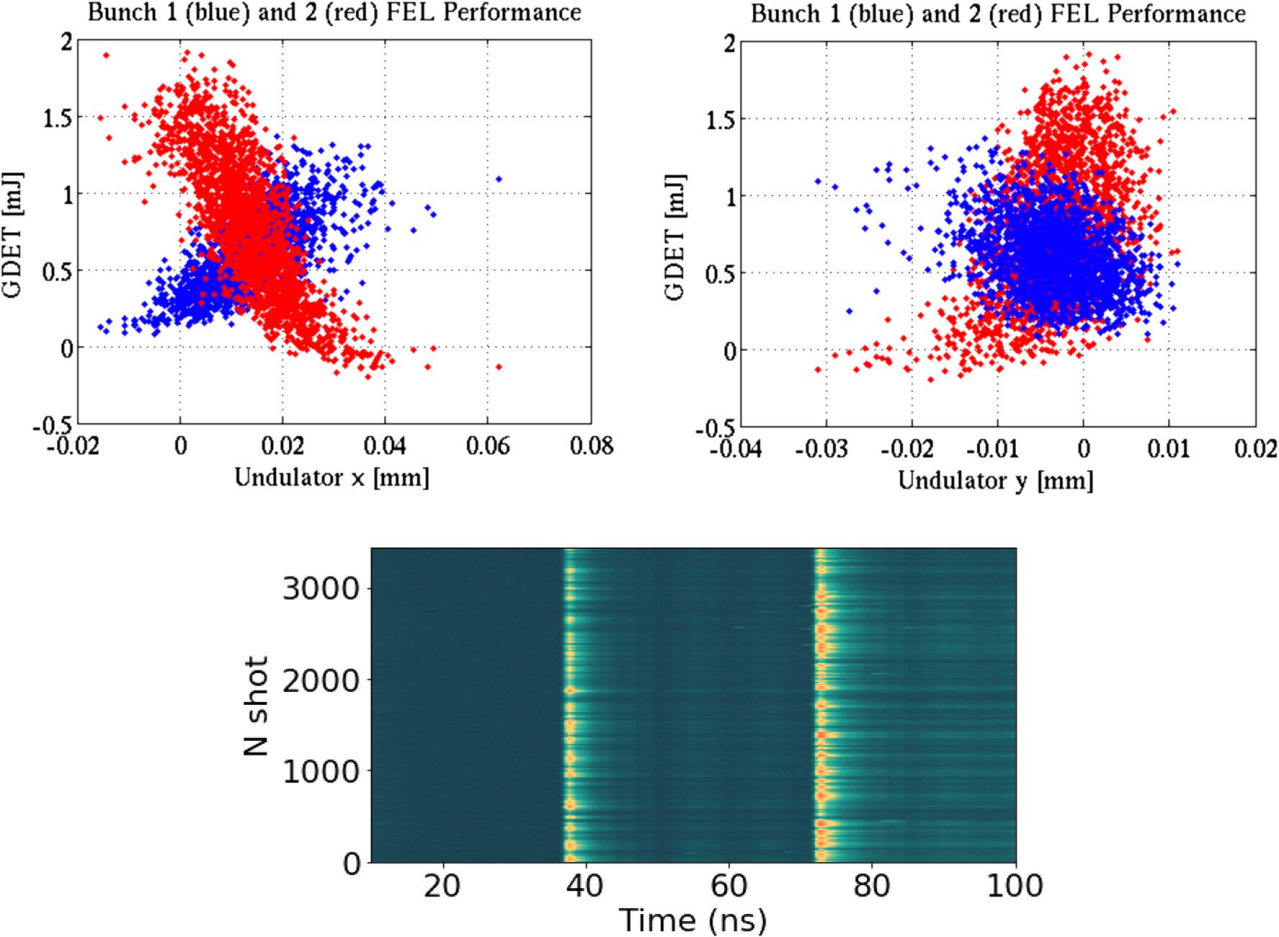
X and Y undulator trajectory displacements in the HXR line for the first and second electron bunches, 35 ns apart, as a function of their XFEL performance (top row). LCLS-HXR double bunch time-resolved profile was registered with a fast rise-time diode (bottom).

Double- and multi-bucket configurations have been successfully reestablished after the LCLS-II undulator upgrade, on both SXR and HXR lines with the copper linac and similar performance to LCLS-I. For instance, two pulses of 9 keV with about 35 ns separation were recently produced, in support of the first XLO experiment. Similarly to LCLS-I, an anti-correlation in double-pulse intensities has been observed; see Fig. 8. The difference in trajectories inside the undulator is attributed to the fact that copper RF structures have slight misalignments and are fed with a non-uniform 825 ns FWHM RF waveform. Recent measurements suggest that most of trajectory difference for long (>100 ns) separations arises in early L1-L2 linac sections^47^. When the trajectory differences are corrected, the new LCLS undulator system will be able to offer tunable double- and multi-bucket x-ray pulse trains from 200 eV to 18 keV.

### Photon pulse tailoring

Pump-probe experiments may require the two pulses to be different in terms of wavelength, intensity and also pointing. As an example, in the study of explosions, it may be interesting to probe the sample at a different location with respect to the transverse coordinate where the pump pulse has excited the system. The photon wavelength is controlled by the electron bunch energy. Therefore to have different wavelengths, the electron bunch energy has to be, to some extent, controlled independently for the two bunches. Electron bunch energy separation between the two bunches is limited by the bunch transport acceptance. In terms of photon energy, a separation of $\sim 3$% is achievable in the Soft x-rays and a separation of $\sim 1$% is achievable at Hard x-rays. For time-delay larger than 8.4 ns, the energy separation can be achieved by placing the electron bunches at different timing with respect to the SLED RF pulses in the late Linac sections. For lower temporal delays, the two bunches should be placed at different phases right at the gun, similarly to the two-bunch operation^15^. In this case, the electron bunches will have different energies along the entire machine, thus preventing horn collimation in the first bunch compressor, and requiring fine tuning if the wavelength separation is changed. The intensity between the pulses can be adjusted in several ways. The drive laser at the cathode can be controlled to modify the charge, and therefore the amount of photons produced by one of the two electron bunches. Alternatively, since the two bunches are not identical, the intensity ratio can be empirically tuned with the transverse electron bunch optics. Finally, the laser heater can be used to increase the gain-length for one of the two pulses by precisely setting the amount of electron bunch energy spread.

**Figure 9:**
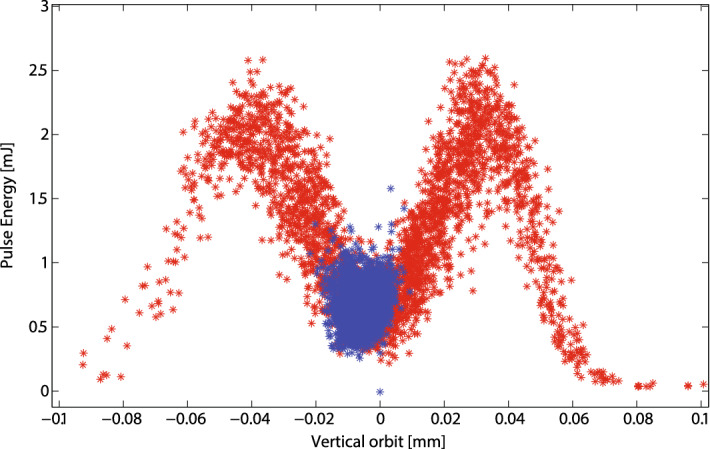
9.5 keV x-ray pulse energies vs largest vertical electrons measured trajectory in the undulator, for a configuration involving two different x-ray pointing directions (LCLS-I). Two different pointings are achieved with the electron bunches travelling on different vertical orbits. For measured orbits of $\sim \pm 40 \mu m$ a single bunch reaches full lasing. For measured orbits close to the undulator axis, both bunches are lasing with a reduction of performance. The red dataset was recorded with a large dispersion of transverse kicks imparted by TCAV3 and shows the full correlation. The blue dataset represents the actual working point with small orbit dispersion.

Two different transverse positions at the target is the most difficult requirement for non split-undulator setups, as an undulator line pointing change will affect both x-ray pulses. To produce such beams in a two-bucket mode, each electron bunch must travel on a different orbit in the undulator line. Particularly, the best condition is with the source at a longitudinal position where the transverse separation between the electron bunches is the largest. If the two bunches have different energies, different orbits can be achieved exploiting energy to orbit coupling in a dispersive section. Alternatively, one could impart a kick with the TCAV3 located downstream from the second bunch compressor. This technique is available also when the bunches have the same energy. Figure 9 shows the total pulse energy for a two-bucket operation at 9.5 keV with respect to the vertical orbit offset measured by a specific BPM toward the end of the undulator line. A vertical time-dependent kick imparted by TCAV3, sets the two electron bunches on different vertical orbits. The red set, collected with a large dispersion of the kicks imparted by the transverse deflecting cavity, shows the correlation between the orbits and the pulse energy. For a measured bunch orbit close to $+35 \mu$m, only a single bunch is lasing at full power. Instead, for a measured bunch orbit close to $-45 \mu$m, only the other bunch is lasing. For measured bunch orbits close to the undulator axis, both bunches are equally lasing but with a reduction of performance close to $75\%$. Samples in blue represent a second dataset collected at the machine working point to produce double pulses with the same average intensity and two separate pointings. The amount of orbit jitter in the figure is representative of what can be achieved with the present copper linac. The x-ray beam pointing difference was tuned to be about 5 times of the RMS e-beam size, with about factor of 6 reduction in XFEL performance.

## Discussion

Double x-ray FEL pulses with long temporal separation ranging from 350 ps to hundreds of nanoseconds in discrete 350 ps steps were demonstrated at LCLS-I and LCLS-II with a copper linac two-bucket scheme. The performance of each pulse can be similar to the one achieved in single bunch operation. Further extension of the scheme up to 8 bunches is currently being developed, by modifying the injector drive lasers to accommodate more electron bunches to be accelerated in different RF buckets. Combination with Split undulator and Fresh-slice could produce pairs of pulses with nanosecond separation, with wavelength control and temporal separation up to 1 ps within each pair. The presented scheme will be necessary for pioneering experiments in many different areas of research, from atomic physics and fluid dynamics to quantum optics, magnetism and topological materials. Two-bucket and multi-bucket schemes pave the way for the next generation x-ray cavity based systems, such as CBXFEL and XLO. The extension of this technique to superconducting linac will be a subject of future studies.
